# Effects of insulin like growth factors on early embryonic chick limb myogenesis

**DOI:** 10.1371/journal.pone.0185775

**Published:** 2017-10-03

**Authors:** Rabeea Hazim Mohammed, Helen Anderton, John Michael Brameld, Dylan Sweetman

**Affiliations:** School of Biosciences, University of Nottingham, Sutton Bonington Campus, Loughborough, United Kingdom; Laboratoire de Biologie du Développement de Villefranche-sur-Mer, FRANCE

## Abstract

Limb muscles derive from p*ax3* expressing precursor cells that migrate from the hypaxial somite into the developing limb bud. Once there they begin to differentiate and express muscle determination genes such as *MyoD*. This process is regulated by a combination of inductive or inhibitory signals including Fgf18, retinoic acid, HGF, Notch and IGFs. IGFs are well known to affect late stages of muscle development and to promote both proliferation and differentiation. We examined their roles in early stage limb bud myogenesis using chicken embryos as an experimental model. Grafting beads soaked in purified recombinant IGF-I, IGF-II or small molecule inhibitors of specific signaling pathways into developing chick embryo limbs showed that both IGF-I and IGF-II induce expression of the early stage myogenic markers p*ax3* and *MyoD* as well as *myogenin*. Their effects on *pax3* and *MyoD* expression were blocked by inhibitors of both the IGF type I receptor (picropodophyllotoxin, PPP) and MEK (U0126). The PI3K inhibitor LY294002 blocked IGF-II, but not IGF-I, induction of p*ax3* mRNA as well as the IGF-I, but not IGF-II, induction of *MyoD* mRNA. In addition SU5402, an FGFR/ VEGFR inhibitor, blocked the induction of *MyoD* by both IGFs but had no effect on *pax3* induction, suggesting a role for FGF or VEGF signaling in their induction of *MyoD*. This was confirmed by in situ hybridization showing that *FGF18*, a known regulator of *MyoD* in limb myoblasts, was induced by IGF-I. In addition to their well-known effects on later stages of myogenesis via their induction of *myogenin* expression, both IGF-I and IGF-II induced *pax3* and *MyoD* expression in developing chick embryos, indicating that they also regulate early stages of myogenesis. The data suggests that the IGFs may have slightly different effects on IGF1R signal transduction via PI3K and that their stimulatory effects on *MyoD* expression may be indirect, possibly via induction of *FGF18* expression.

## Introduction

During development the limb muscles are derived from *pax3* expressing cells from the hypaxial region of somites. These cells delaminate and migrate into the limb buds where they begin to differentiate and express muscle specific markers such as members of the Myogenic Regulatory Factor (MRF) family of transcription factors [1–5]. The migration of these cells is induced by CXCR4 [6, 7] and HGF [8–10], which also acts to prevent premature differentiation of these cells. The majority of the migratory cells will contribute to muscle although some will also become endothelial cells [11]. Once in the limb, the myogenic precursors form the dorsal and ventral muscle masses and begin to differentiate, a process regulated by the induction the MRFs; first myoblasts express *Myf5*, then *MyoD*, *myogenin* and finally *MRF4* [12].

Numerous signaling molecules regulate the differentiation of the limb myoblasts. Their differentiation is inhibited by sonic hedgehog [13] and BMP [14], promoted by FGFs, such as FGF18 [15, 16], while other molecules can act to either block or induce myogenic genes depending on the stage of development and concentration, such as retinoic acid [16, 17].

The insulin like growth factors, IGF-I and IGF-II, are well characterized promoters of muscle growth in development [18], including in chicken embryos [19]. They act through the IGF type 1 receptor in muscle growth and regeneration [20] primarily by promoting the AKT/mTOR and MAPK signaling pathways [21–23].

During limb development several components of the IGF signaling machinery are expressed [24] and IGF signaling regulates the formation of the limb skeleton [25]. Retroviral overexpression of IGF-I in limbs also increases muscle size by promoting myoblast proliferation, leading to increased numbers of muscle fibres [19], and in ovo injection of IGF-I can have effects lasting into adulthood [26]. However, as well as promoting proliferation, IGFs can also induce *myogenin* expression [27] and it is clear that they have a complex role in developing muscle.

To try and understand the effects of IGFs during early embryonic myogenesis we used the chicken embryo limb bud as a model [28, 29] by grafting beads soaked in purified growth factors or other signaling inhibitory molecules at defined stages of embryogenesis to determine their effects on myogenesis. Here we show that grafting IGF beads into early developing chicken embryo limbs induces the expression of *pax3*, a marker of proliferative muscle precursor cells, while later grafting also induces both *MyoD* and *myogenin*, which are associated with the early and late stages of myogenic differentiation respectively. Using various inhibitors we show that the effects on both *pax3* and *MyoD* require MEK signaling while *MyoD* induction is dependent on secondary signaling through either FGFs or VEGF; in addition we show that IGF-I can induce *FGF18* expression in limb buds. A PI3K inhibitor produced a more complex picture with different effects depending on whether the limbs were treated with IGF-I or–II.

## Materials and methods

### Growing and staging of experimental animals

Fertilized white leghorn chicken (Gallus gallus) eggs were purchased from Henry Stewart Limited (Norwich, UK). Eggs were incubated at 15°C for up to 5 days until the day of use then transferred to 38°C (Forma scientific CO2 water incubator) until they reached the required stages of development. Embryos were staged according to Hamburger and Hamilton [30].

### IGF and pharmacological inhibitor beads

Heparin beads (Sigma H-5263) were soaked in recombinant human IGF-I or IGF-II (Peprotech) at 1mg/ml in phosphate buffered saline (PBS) with 0.1% Bovine Serum Albumin (BSA). AG 1-X2 beads (BioRad) were incubated in Picropodophyllotoxin (PPP, Tocris Bioscience), U0126 (Cell Signaling), LY 294002 (Calbiochem) or SU5402 (Calbiochem), all reconstituted in DMSO at 10mM. Beads were incubated for at least one hour in the dark before being washed briefly in 2% phenol red and rinsed in PBS before grafting. Beads were grafted into limb buds with a sharpened tungsten needle, resealed with sellotape and reincubated for 18-48h as described previously [31].

### In situ hybridization

In situ hybridization was performed as described previously [12]. Embryos were collected, staged [30], fixed in 4% paraformaldehyde (PFA) at 4°C overnight, washed in 50% methanol/PBS with 0.1% Tween (PBSTw) then dehydrated by washing twice in 100% methanol. Embryos were then stored at -20°C.

Embryos were rehydrated in a series of 75%, 50% and 25% methanol/PBSTw then washed twice in PBSTw. Embryos older than HH stage 20 were treated with proteinase K in PBSTw at 10 μg/mL for 25 min, rinsed twice in PBSTw then post-fixed in 4% PFA/0.1% glutaraldehyde for 20 min at room temperature followed by two washes in PBSTw. Embryos were then washed in 1: 1 PBSTw:hyb solution (50% formamide, 1.3xSSC pH 5, 5 mM EDTA, 50 μg/mL yeast RNA, 0.2% Tween-20, 0.5% CHAPS, 100 μg/mL heparin), washed with hyb solution for 10 min, then incubated in fresh hyb solution at 65°C for at least 2 h. Probes were added in pre-warmed hyb solution at 65°C were added and incubated overnight at 65°C. Embryos were rinsed twice in hyb solution at 65°C, washed for 10 min in hyb solution at 65°C, then washed twice for 30 min in washing buffer (50% formamide, 1xSSC pH 5, 0.1% Tween-20) at 65°C. Embryos were washed for 10 min at 65°C in 1:1 washing buffer:MABT (100 mM maleic acid, 150 mM NaCl, 0.1% Tween-20, pH 7.5), rinsed three times in MABT, and washed twice for 30 min in MABT. They were then blocked in 2% Roche blocking reagent (cat no. 11096176001) in MABT for 1 h. Anti-Dig-AP Fab fragments (Roche, cat no. 11093274910) were diluted 1:2000 in 2% Roche blocking reagent in MABT and incubated overnight at 4°C. Embryos were washed three times for 1 h in MABT and then twice for 10 min in NTMT (100 mM NaCl, 100 mM Tris pH 9.5, 50 mM MgCl_2_, 1% Tween-20). Colour was developed with 9μg NBT (4-nitro blue tetrazolium chloride at 75 mg/ml in 70% dimethylformamide) and 7μl BCIP (5-bromo-4-chloro-3-indolyl-phosphate, 4-toluidine salt at 50 mg/ml in dimethylformamide) per ml of NTMT. After the staining reaction, embryos were de-stained in high detergent mix, 5xTBST (for 100 mL of a 5xsolution: 4 g NaCl, 12.5 mL 1 M Tris-HCl pH 7.5, 0.1 g KCl, 5 mL Tween-20) to reduce background and, if required, re-stained. Stained embryos were stored in 4% PFA with 0.05% sodium azide prior to imaging. Fixed embryos were imaged using a Leica DFC320 camera on a Leica MZ10F stereomicroscope with Leica Acquisition Suite software. MRF and *Fgf18* probes were made as described previously [16] as was the *pax3* probe [32].

### Paraffin embedding and sectioning of chick embryos

Embryos were washed twice in 1X PBS at RT and in sterile distilled water then Dehydrated through an ethanol series of 25%, 50%, 70%, 90% and 100% ethanol. Embryos were cleared in xylene then transferred into hot paraffin wax at 65°C for 2 hours. Embryos were orientated in paraffin wax solution and cooled for 2 hours at 4°C. Sections were cut at 7 μm thickness on a HM 355 microtome, placed onto glass microscope slides then mounted in Omnimount (Histological Mounting Medium HS-110). Slides were photographed on an Olympus BH-2 microscope.

## Results

**Fig 1:**
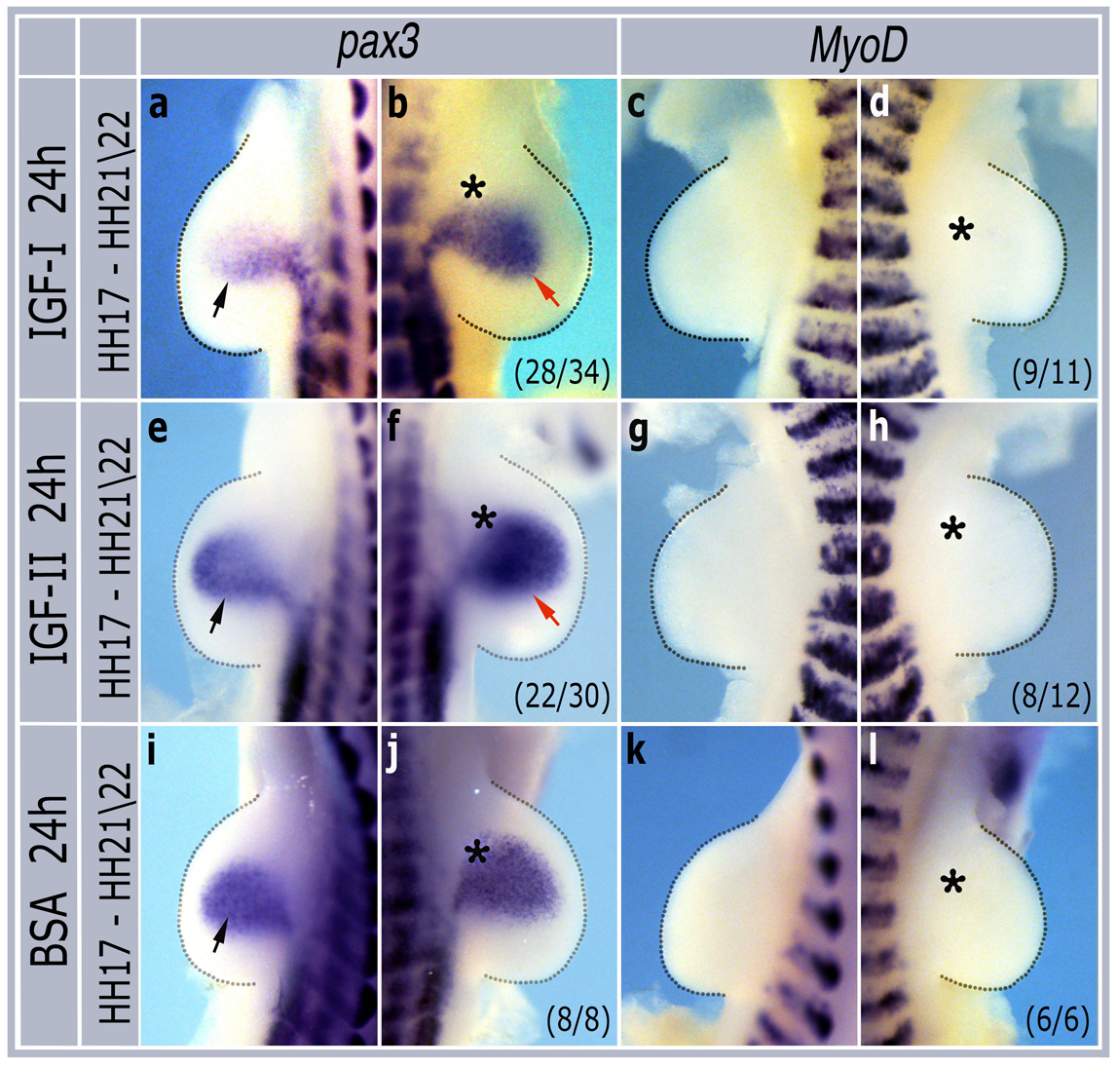
Grafting IGF beads at HH stage 17 induces *pax3* but not *MyoD* expression. Contralateral pairs of forelimbs grafted with IGF-I (a,b,c,d), IGF-II (e,f,g,h) or BSA (i,j,k,l), incubated for 24h and then harvested for in situ hybridization with *pax3* or *MyoD* probes. Asterisks show positions of grafted beads. Black arrows indicate normal expression domains in non-grafted limbs while red arrows show induced expression. Numbers in brackets indicate the numbers of similar embryos out of the total number analysed.

To determine their effects on limb bud myogenesis, heparin beads soaked in either IGF-I or IGF-II were grafted into developing limb buds in ovo at HH stage 17 and incubated for 24 hours until they had reached HH stage 21/22. In situ hybridization showed a clear upregulation of *pax3* mRNA in grafted limbs by both IGF-I (28/34 embryos, Fi g1 a,b) and IGF-II (22/30 embryos, Fig 1e and 1f). In these embryos *MyoD* was only rarely upregulated by either IGF-I (2/11 embryos, Fig 1c and 1d) or IGF-II (4/12 embryos, Fig 1g and 1h) after 24 hours. Control beads soaked in BSA had no effect on either *pax3* (8/8 embryos, Fig 1I and 1j) or *MyoD* (6/6 embryos, Fig 1k and 1l) expression.

**Fig 2:**
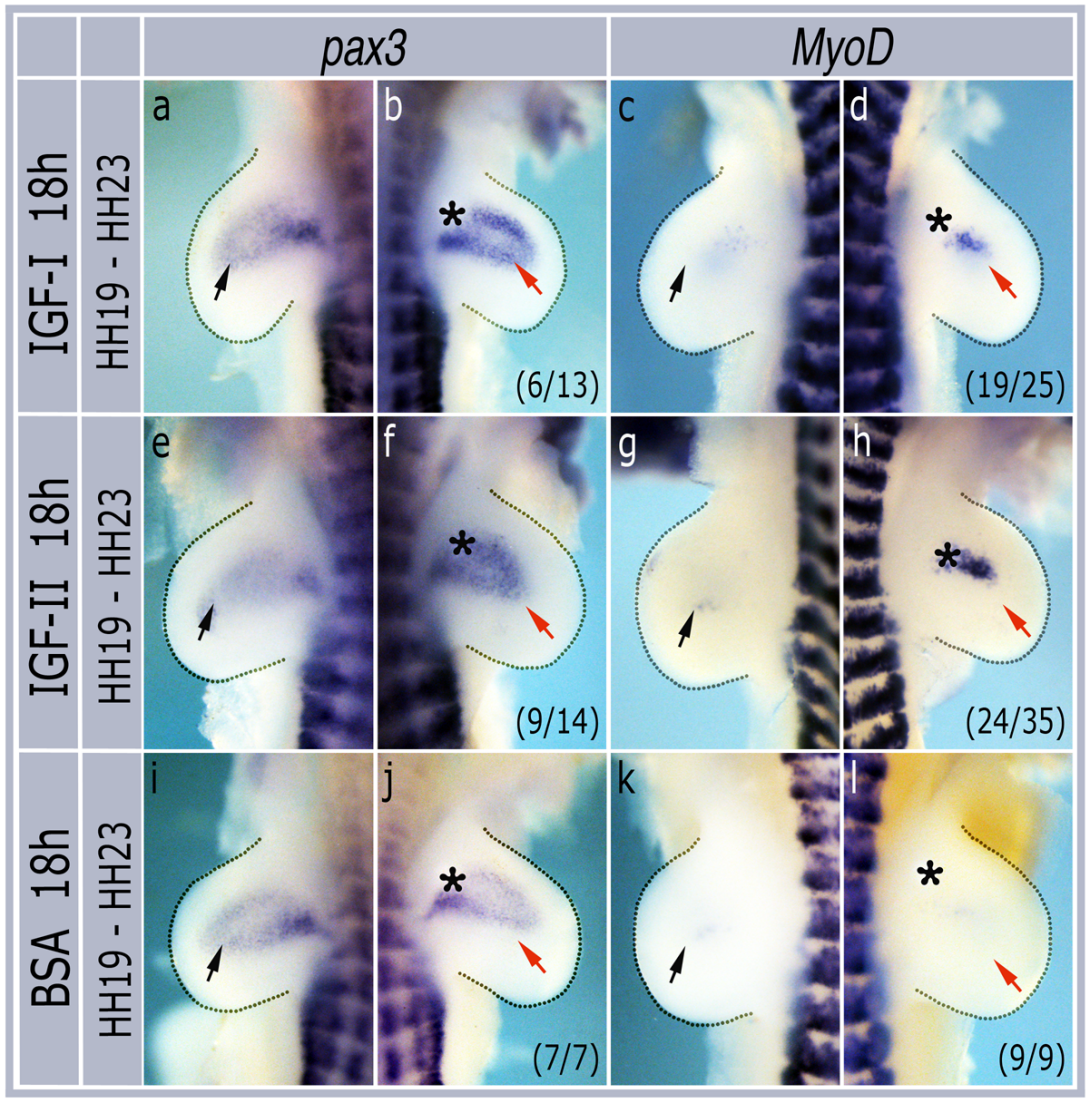
Grafting IGF beads at HH stage 19 induces *pax3* and *MyoD* expression. Contralateral pairs of forelimbs grafted with IGF-I (a,b,c,d), IGF-II (e,f,g,h) or BSA (i,j,k,l), incubated for 18h and then harvested for in situ hybridization with *pax3* or *MyoD* probes. Asterisks show positions of grafted beads. Black arrows indicate normal expression domains in non-grafted limbs while red arrows show induced expression. Numbers in brackets indicate the numbers of similar embryos out of the total number analysed.

To see if these genes were induced by IGFs at later stages of development heparin beads soaked in either IGF-I or IGF-II were grafted into developing limb buds at HH stage 19 and incubated for 18 hours until they had reached HH stage 23. In these embryos IGF-I induced both *pax3* (6/13 embryos, Fig 2a and 2b) and *MyoD* (19/25 embryos, Fig 2c and 2d) expression. Similarly, IGF-II also induced both *pax3* (9/14 embryos, Fig 2e and 2f) and *MyoD* (24/35 embryos, Fig 2g and 2h), whereas BSA control beads had no effect on either *pax3* (7/7 embryos, Fig 2i and 2j) or *MyoD* (9/9 embryos, Fig 2k and 2l) expression.

**Fig 3:**
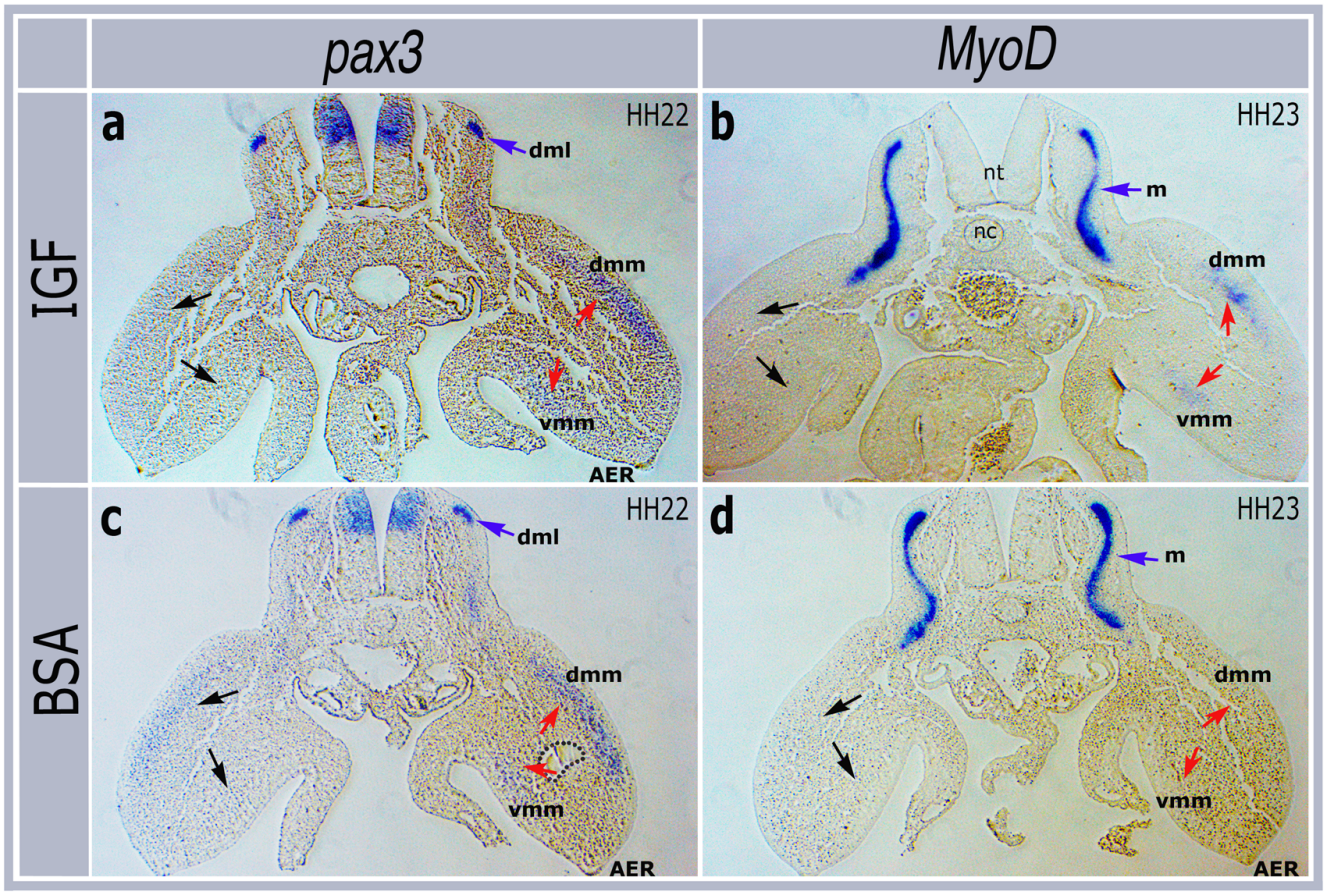
IGF-I induced expression of *pax3* (a) and *MyoD* (b) is confined to dorsal and ventral muscle masses. BSA beads (c,d) do not affect normal expression of these genes. dml: dorso-medial lip, dmm: dorsal muscle mass, vmm, ventral muscle mass, nt: neural tube, nc, notochord, m: myotome, AER: apical ectodermal ridge. Red arrows show regions of increased mRNA expression in grafted limbs, black arrows the regions of endogenous gene expression in the contralateral limbs.

To confirm that the induced expression of both *pax3* and *MyoD* was in myogenic progenitors we cut transverse sections of manipulated embryos. These showed that upregulation of *pax3* and *MyoD* mRNA was observed in the dorsal and ventral muscle masses (Fig 3a and 3b), the regions of the limb buds where myogenic differentiation normally occurs. In contrast the non-manipulated limbs and other expression domains, such as the dorsal neural tube and dorso-medial lip of the somite, which express *pax3*, and the myotome, which expresses *MyoD*, remained unaffected (Fig 3c and 3d).

**Fig 4:**
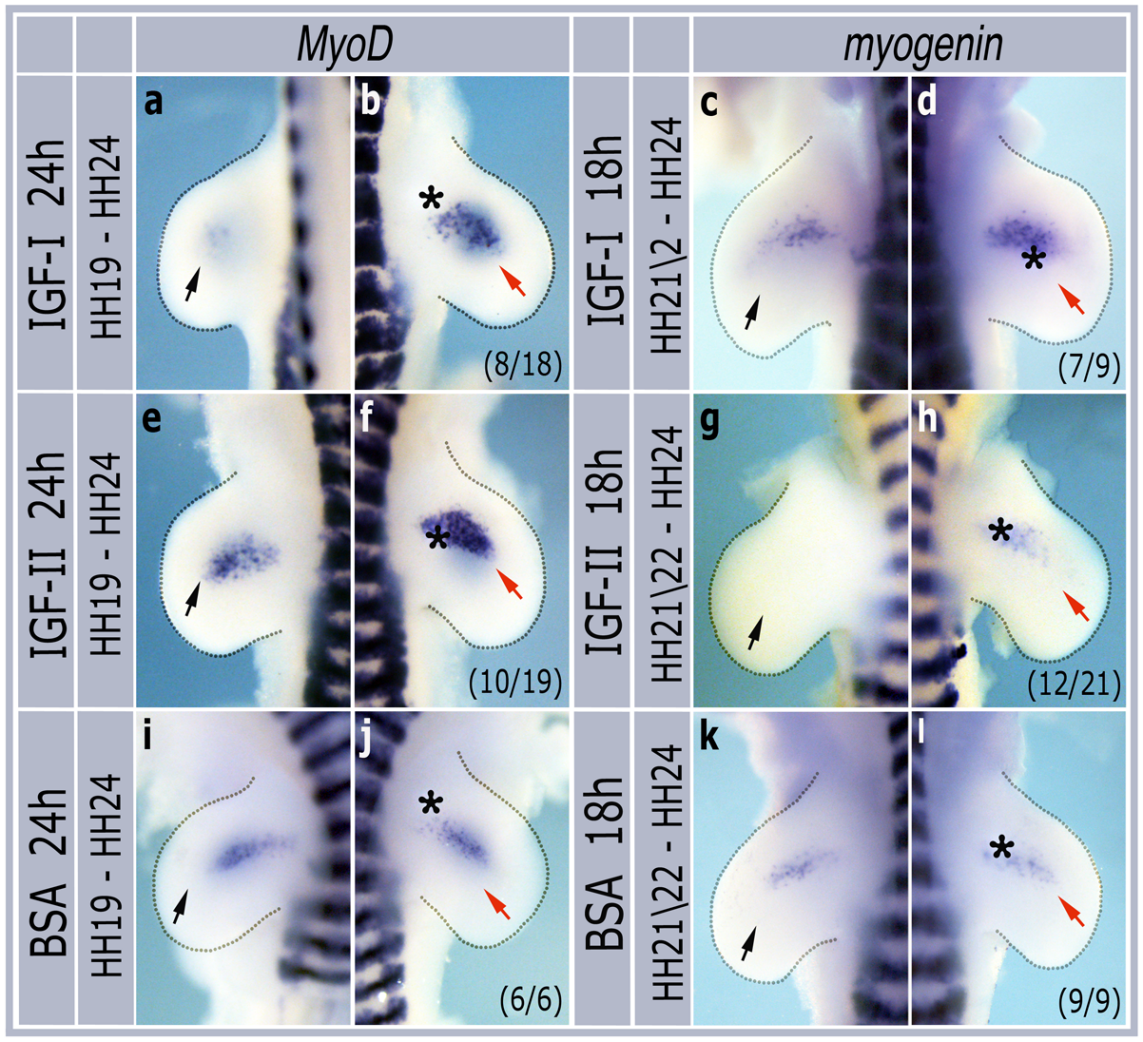
IGF-1 and–II induce *MyoD* and *myogenin* in HH stage 24 limbs. Contralateral pairs of forelimbs grafted with IGF-I (a,b,c,d), IGF-II (e,f,g,h) or BSA (i,j,k,l), incubated for 24h and then harvested for in situ hybridization with *MyoD* or *myogenin* probes. Asterisks show positions of grafted beads. Black arrows indicate normal expression domains in non-grafted limbs while red arrows show induced expression. Numbers in brackets indicate the numbers of similar embryos out of the total number analysed.

To see if IGFs could also induce expression of later myogenic markers we incubated embryos following bead grafts to HH stage 24, the point at which myogenic cells begin to express markers of terminal differentiation such as *myogenin*. IGF-I beads grafted at HH stage 19 and incubated for 24h to reach HH stage 24 induced *MyoD* expression (8/18 embryos, Fig 4a and 4b), while beads grafted at HH stage 21/22 and incubated for 18h to reach HH stage 24 induced *myogenin* (7/9 embryos, Fig 4c and 4d). Similar effects were seen with IGF-II beads which induced both *MyoD* (10/19 embryos, Fig 4e and 4f) and *myogenin* (12/21 embryos, Fig 4g and 4h) at HH stage 24. In contrast BSA beads had no effect on either *MyoD* (6/6 embryos, Fig 4i and 4j) or *myogenin* (9/9 embryos, Fig 4k and 4l) expression.

**Fig 5:**
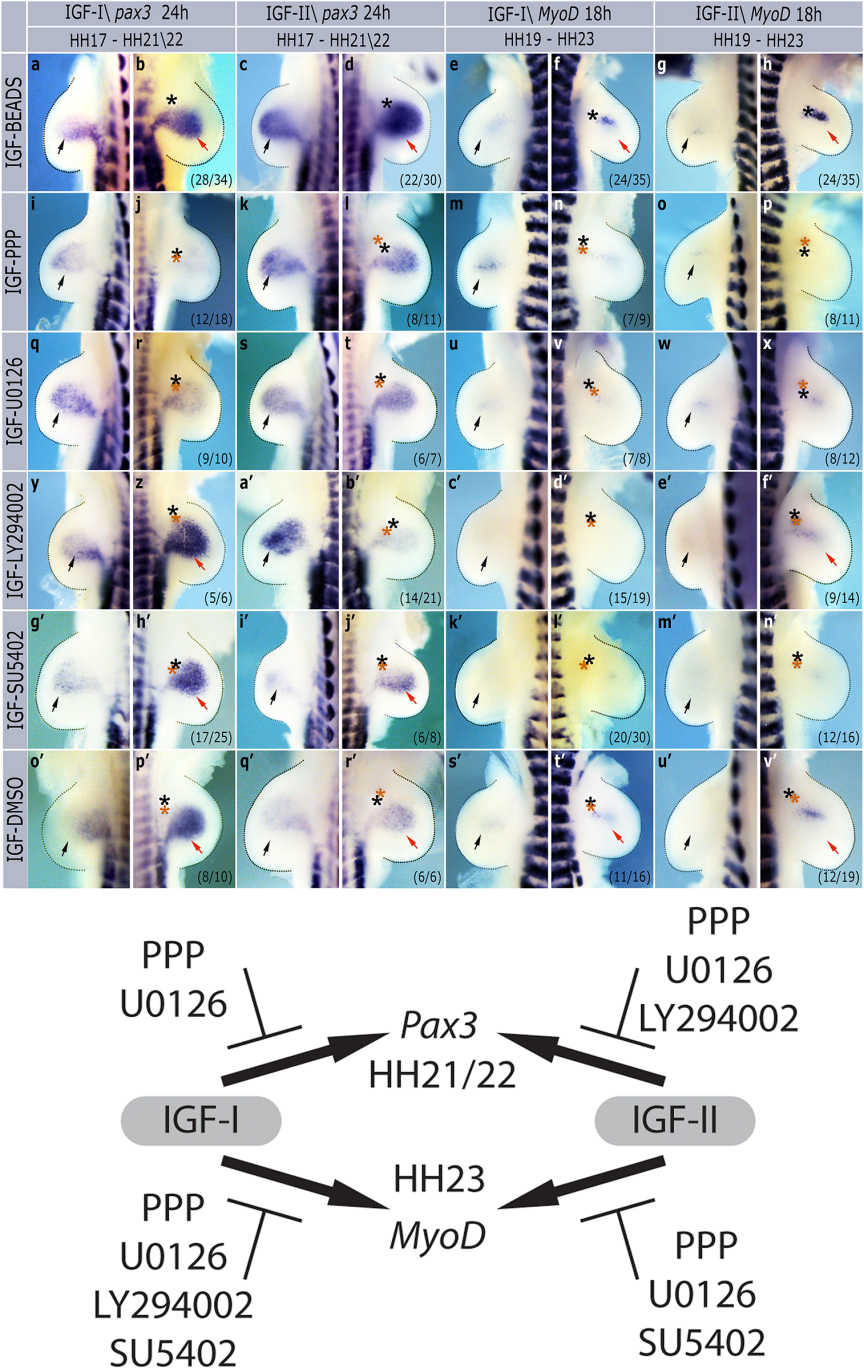
Effects of small molecule inhibitors on *pax3* and *MyoD* induction by IGFs. Contralateral pairs of forelimbs grafted with IGF-I or IGF-II beads along with small molecule inhibitors, incubated for 24 or 18h and then harvested for in situ hybridization using *pax3* or *MyoD* probes. Black asterisks show positions if IGF beads, red asterisks inhibitor beads. Black arrows show normal expression domains in non-grafted limbs, red arrows induced expression by IGF. Numbers in brackets indicate the number of similar embryos out of total number of grafts. A summary diagram of which molecules inhibited IGF-I and/or IGF-II induction of *pax3* and *MyoD* mRNA is also included.

To investigate the downstream signaling pathways mediating IGF induction of *pax3* and *MyoD* we co-grafted IGF beads with beads soaked in small molecule signaling inhibitors. We tested these signal transduction inhibitor effects on *pax3* induction following IGF beads grafted at HH stage 17 (Fig 5a, 5b, 5c and 5d) and *MyoD* induction after grafting at HH stage 19 (Fig 5e, 5f, 5g and 5h). Results are shown in Fig 5 and summarized in Table 1.

**Table 1:** Summary of effects of small molecule signaling inhibitors on IGF-I and IGF-II induction of *pax3* and *MyoD* expression in developing limb of chick embryos.

Graft at HH 17, incubate for 24h	*pax3* induction		Graft at HH 19, incubate for 18h	*MyoD* induction	
IGF-I	28/34	82%	IGF-I	19/25	76%
IGF-I + PPP	6/18	33%	IGF-I + PPP	2/9	22%
IGF-I + U0126	1/10	10%	IGF-I + U0126	1/8	12.5%
IGF-I + LY294002	5/6	83%	IGF-I + LY294002	4/19	21%
IGF-I + SU5402	17/25	68%	IGF-I + SU5402	10/30	33%
IGF-I + DMSO	8/10	80%	IGF-I + DMSO	11/16	69%
Graft at HH 17, incubate for 24h	*pax3* induction		Graft at HH 19, incubate for 18h	*MyoD* induction	
IGF-II	22/30	73%	IGF-II	24/35	69%
IGF-II + PPP	3/11	27%	IGF-II + PPP	3/11	27%
IGF-II + U0126	1/7	14%	IGF-II + U0126	4/12	33%
IGF-II + LY294002	7/21	33%	IGF-II + LY294002	9/14	64%
IGF-II + SU5402	6/8	75%	IGF-II + SU5402	4/16	25%
IGF-II + DMSO	6/6	100&	IGF-II + DMSO	12/19	63%

To confirm that the IGFs were acting through the IGF type 1 receptor we co-grafted IGFs with picropodophyllotoxin (PPP), a specific inhibitor of IGF1R autophosphorylation. PPP beads blocked IGF-I induction of both *pax3* (12/18 embryos, Fig 5i and 5j) and *MyoD* (7/9 embryos, Fig 5m and 5n) as well as IGF-II induction of *pax3* (8/11 embryos, Fig 5k and 5l) and *MyoD* (8/11 embryos, Fig 5o and 5p).

We then tested U0126, a MEK inhibitor, in the same assay. U0126 beads effectively blocked IGF-I induction of *pax3* (9/10 embryos, Fig 5q and 5r) and *MyoD* (7/8 embryos, Fig 5u and 5v) as well as IGF-II induction of *pax3* (6/7 embryos, Fig 5s and 5t) and *MyoD* (8/12 embryos, Fig 5w and 5x).

IGF beads were also co-grafted with beads soaked in LY294002, a PI3K inhibitor. In this case IGF-II induction of *pax3* was blocked (14/21 embryos, Fig 5a’ and 5b’) but IGF-I induced *pax3* expression was not affected (5/6 embryos, Fig 5y and 5z). In contrast, IGF-I induction of *MyoD* was blocked by LY29004 beads (15/19 embryos, Fig 5c’ and 5d’) but there was no effect of LY294002 beads on IGF-II induction of *MyoD* (9/14 embryos, Fig 5e’ and 5f’).

Initially as a control we also tested an inhibitor of the FGFRs and VEGFR, SU54502. As expected this has no effect on IGF-I (17/25 embryos, Fig 5g’ and 5h’) or IGF-II (6/8 embryos, Fig 5i’ and 5j’) induction of *pax3* but, unexpectedly, it inhibited *MyoD* induction by both IGF-I (20/30 embryos, Fig 5k’ and 5l’) and IGF-II (12/16 embryos, m’,n’).

As another control we also tested beads soaked in DMSO, the solvent used for these inhibitors. DMSO had no effect on *pax3* or *MyoD* induction by either IGF-I or IGF-II (Fig 5o’, 5p’, 5q’, 5r’, 5s’, 5t’, 5u’ and 5v’).

**Fig 6:**
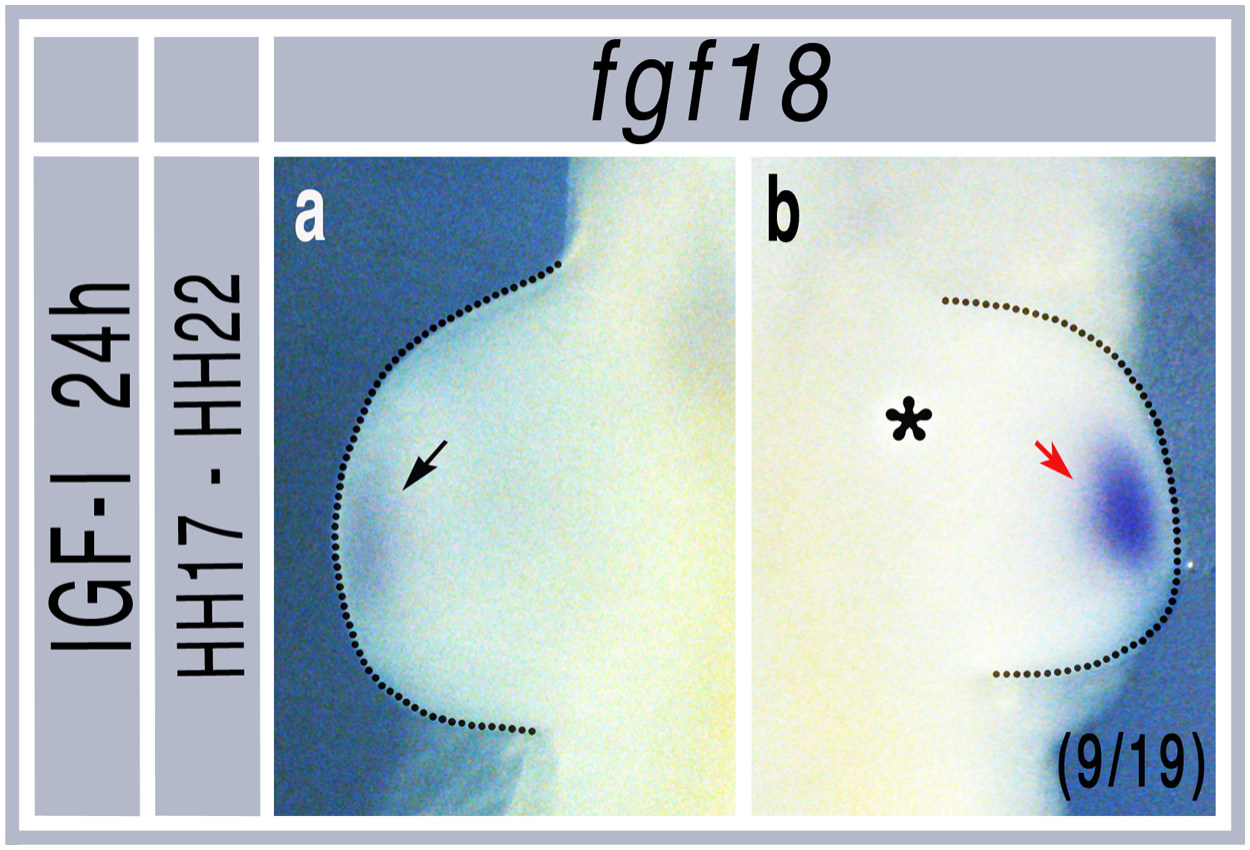
Increased *Fgf18* expression following IGF-I bead grafts. a) non-grafted limb showing normal expression domain of *Fgf18* at HH stage 22. b) increased *Fgf18* following IGF-I bead grafting. Asterisk shows position of bead. Red arrow shows region of increased *Fgf18* mRNA. Numbers in brackets indicate the number of similar embryos out of total number of grafts.

The ability of SU5402, an FGFR and VEGFR inhibitor, to block *MyoD* induction by IGFs was unexpected. One possible explanation was that this was an indirect effect caused by IGF induced upregulation of FGF. To test this we grafted IGF-I beads at HH stage 17, incubated embryos for 24h to HH stage 22 and then measured their effect on *Fgf18* mRNA, which is known to induce *MyoD* expression in limb bud myogenic precursors. In 9/19 embryos we saw increased levels of *Fgf18* in grafted limb buds (Fig 6).

## Discussion

The specification and differentiation of muscle cells provides an excellent paradigm to examine inductive events during development. As *pax3* expressing precursor cells migrate into limb buds they begin to differentiate by expressing *MyoD* and, subsequently, *myogenin* in response to a range of signals [12, 33].

Although IGFs are well known to regulate muscle formation in embryos and muscle growth in adult animals [34–36] there remain many unanswered questions about their roles; for example how they are able to promote both proliferation and differentiation of myoblasts, behaviours that should be mutually exclusive. Here we use the chicken embryo model to examine some of the signaling events that underlie these activities by grafting beads soaked in IGF-I or IGF–II along with specific signaling inhibitors.

In early limb buds (HH stage 17) both IGFs were able to induce upregulation of *pax3* mRNA but not *MyoD*, while in slightly later limbs (HH stage 19) both *pax3* and *MyoD* mRNA were induced. We have previously shown that these early myogenic progenitors are resistant to signals that promote differentiation and that this is mediated by retinoic acid [16]. The data we present here is consistent with the idea that signals in the early limb prevent premature differentiation of muscle cells, presumably to ensure that there are sufficient precursors produced to contribute to the muscles.

The ability of IGFs in older embryos to induce *pax3* expression, a marker of proliferative precursors, and *MyoD* and *myogenin*, markers of early and late stages of differentiation, is less easy to explain although this is a commonly observed feature of these molecules [34]. There are several models that could explain this apparent paradox.

The increase in *pax3* expression could be due to (i) increased proliferation of the migratory precursors, (ii) higher numbers of cells migrating from the hypaxial somite into the limbs or (iii) recruitment of additional cells from within the limb to the myogenic lineage. As limb bud mesenchyme cells do not contribute to muscle [37, 38] or express *pax3* [39, 40] it is unlikely that these cells are being respecified and therefore the recruitment (iii) model is unlikely. It is also possible, given that in situ hybridization does not provide single cell resolution, that the increase in *pax3* levels indicates higher transcription of *pax3* mRNA within the same number of cells. It is also important to bear in mind that we cannot exclude indirect effects on *pax3* expression; for example IGFs could be inducing expression of other signaling molecules, such as HGF, that are known to enhance myoblast proliferation [8–10].

The induction of *MyoD* and *myogenin* are harder to explain in the context of increased p*ax3* expression. It is possible that this is a stochastic effect because there are more progenitors in the limb; increased *MyoD* and *myogenin* levels are observed simply because there are more myogenic cells differentiating. An alternative explanation is that IGF signaling has different effects at different stages of development and differentiation. This is consistent with the differences seen at HH stages 17 and 19. At earlier stages myogenic precursors are not competent to induce *MyoD*, either because of their epigenetic state or because of high levels of inhibitors in the proximal limb, such as retinoic acid [16]. In later embryos RA levels have declined and the cells will also have moved further along the differentiation process, potentially making them able to respond to IGFs in a different way. One other possible explanation is that the IGFs will also affect the surrounding limb bud mesenchyme, changing the signaling environment of the cells. Support for this model comes from the surprising observation that *MyoD* induction by IGFs is blocked by an inhibitor of the FGF and VEGF receptors. In this model IGFs act indirectly by the induction of pro-myogenic signals, such as *FGF18*, in the surrounding tissues. We have shown previously that beads soaked in FGF18 can induce *MyoD*, but not *pax3*, expression in developing limbs at these stages of development [16] and the induction of *Fgf18* mRNA by IGF-I, as well as the ability of SU5402 to block IGF induced *MyoD* expression, is consistent with this model. However, as this inhibitor also blocks signaling though VEGFR2, which is known to affect myogenesis, it is also possible that IGFs induce members of the VEGF family and these could also contribute to induction of MyoD.

Further complexity in the responses of limb bud myoblasts to IGFs is apparent when they are exposed to a variety of signal transduction inhibitors. *pax3* and *MyoD* induction in response to IGF-I and–II were both blocked by PPP, an IGF1R inhibitor, and U0126, which blocks MEK activity and so prevents ERK phosphorylation. Surprisingly, given the very well characterized links between IGFs and PI3K/AKT/mTOR signaling, the PI3K inhibitor LY294002 specifically blocked IGF-I induced *MyoD* expression and IGF-II induced *pax3* expression. This could be because, in these embryos, the IGF-I and -II are interacting with other receptors, for example the insulin receptor to trigger this pathway [41]. However, this is hard to reconcile with the data showing that PPP can block all these responses.

In summary our data show limb bud muscle precursors at HH stage 17 respond to IGF signaling by upregulating *pax3*, a marker of early proliferating muscle precursor cells and that later, at HH stage 19, they also upregulate later markers of differentiation, *MyoD* and *myogenin*. All these events are controlled by the IGF1R receptor, which is expressed throughout the limb buds at these stages [24], involving signaling through the ERK MAPK pathway. IGF-I and–II appear to have differential effects through PI3K signaling at HH stages 17 and 19 while the induction of *MyoD* is, at least in part, dependent on FGF receptors, possibly through induction of *FGF18* in the limb bud mesenchyme.

## Ethical approval

All experiments were completed before 14 days of incubation, two thirds of the way through chicken embryo development. Therefore embryos used in this project does are not regulated under the UK Animals (Scientific Procedures) Act of 1986. All procedures were discussed and agreed with the University of Nottingham ethics officer. Fertilised eggs were purchased from reputable commercial suppliers (Henry Stewart) who specialise in providing eggs for research. Their farms are registered under DEFRA’s Poultry Health Scheme which ensures disease control programmes that are up to the latest EU standards. They also comply with RSPCA’s Freedom Food Code of Practice promoting the ‘Five Freedoms’ for best practice welfare.
