# Correction: Effects of insulin like growth factors on early embryonic chick limb myogenesis

**DOI:** 10.1371/journal.pone.0189395

**Published:** 2017-12-05

**Authors:** Rabeea Hazim Mohammed, Helen Anderton, John Michael Brameld, Dylan Sweetman

The following information is missing from the Funding section: This work was supported by the Biotechnology and Biological Sciences Research Council (grant BB/J014508/1).
